# Time to prioritise the use of participatory research methods for people with intellectual disabilities

**DOI:** 10.1192/bjp.2025.96

**Published:** 2025-05-21

**Authors:** Madiha Majid, Olamide Todowede, Ashok Roy, Gerald Jordan, Stefan Rennick-Egglestone

**Affiliations:** School of Health Sciences, Institute of Mental Health, University of Nottingham, Nottingham, UK; Coventry and Warwickshire Partnership Trust, Coventry, UK; School of Health Sciences, Institute of Mental Health, University of Nottingham, Nottingham, UK; Coventry and Warwickshire Partnership Trust, Coventry, UK; University of Birmingham, College of Life and Environmental Science, School of Psychology, Institute of Mental Health, Centre for Urban Wellbeing, Birmingham, UK; School of Health Sciences, Institute of Mental Health, University of Nottingham, Nottingham, UK; NIHR Nottingham Biomedical Research Centre, Institute of Mental Health, Mental Health & Clinical Neurosciences, University of Nottingham, Innovation Park, Triumph Road, Nottingham, UK

**Keywords:** Intellectual disability, Qualitative research, Mental health services, Human rights, Ethics

## Abstract

People with intellectual disability experience significant health inequality, and consequent poor health outcomes. Research can facilitate change, however there is a risk of researchers propagating inequity by selecting methods that exclude people with some forms of intellectual disability. We argue for participatory research methods that enable inclusion.

Intellectual disability (ID) affects approximately 1.5 million people in the UK. People with ID have poorer health outcomes and shorter life expectancies when compared to the general population(1), including due to significant physical health issues that contribute to premature mortality, such as epilepsy. For people with ID, mental health disorders are more common compared to the general population, and the co-occurrence of other neurodevelopmental diagnoses is high. People with ID experience substantial health inequality. This has been evidenced through the COVID-19 pandemic, where health inequalities were exacerbated and people with ID were more likely to be admitted to hospital and die from COVID-19.

Successive reports and inquiries have sought to understand the health inequalities this population experiences, and their causes. *Death by Indifference* highlighted how institutional injustice through discrimination, poor access to primary care, poor quality care in hospitals and lack of training of health professionals contributed to preventable deaths. The *Michael Inquiry* emphasised the failure of services to make reasonable adjustments for people with ID, and described how this contributed to avoidable ill-health and poor outcomes. The *Confidential Inquiry into Premature Deaths of People with Learning Disabilities* further called into question the quality of health and social care, identifying delays in diagnosis and treatment. In response, the *Learning from Lives and Deaths Report* national programme, reviewed the lives and deaths of people with ID and autism. Their work is informed by a team of individuals with ID, which also helps raise awareness of their findings, with ‘opportunities to empower people with ID to advocate for change’(1) that is clearly needed.

There have been significant concerns surrounding the care of people with ID who also have mental health conditions, additional neurodevelopmental differences, or behaviours that challenge. Through the Winterbourne View Hospital scandal of 2011, the physical and psychological abuse of people with ID and autism at this hospital were exposed. A subsequent national review of services identified that people with ID and autism were inappropriately being admitted to inpatient units due to inadequate community services and often faced lengthy admissions. There were also concerns around the quality of person-centred care plans. The Transforming Care programme sought to address these challenging findings by creating change across all healthcare provision.(2) Subsequently, the provision of more appropriate community care has led to fewer hospital admissions.

Despite some improvement to mental health services for people with ID, Baroness Hollin’s 2023 report reviewing the care of those detained in mental health and specialist ID hospitals still describes ‘*heart-breaking’* care and outcomes for people with ID. This includes psychiatric admissions retraumatising patients through not meeting sensory and communication needs.(3) The report concludes that mental health services continue to contribute to the dehumanisation of people with ID.(3) The current healthcare system is not designed to meet the care needs of people with ID and exacerbates barriers to care.(4) Psychiatrists and other healthcare professionals working with people with ID understand the unique challenges that the population they care for experience, and the systemic problems that limit the type and standard of care that we can provide. From an ethical perspective, we cannot claim to be providing just care in an unjust system where huge disparities exist; much change is needed before truly *transforming care*.

Research is vital to inform evidence-based practices in psychiatry, as knowledge produced by researchers can have a critical influence on changes to policy, practices and services. However, a random sample of 180 NIHR-funded studies conducted in 2019-20 found that 131 effectively excluded people with ID, including due to problems with research design, inadequate researcher training, and capacity and consent procedures.(5) Included studies were assessed by a panel of 25 people with ID. Methods such as interviews or focus groups can exclude some people with ID since they require face-to-face interactions with “in the moment” verbal responses. Questionnaires and surveys will be limited to those with certain literacy and comprehension levels, or their completion dependent on support from carers or support workers. Researchers should consider the potential risks and harms to participants as a result of their research designs. It is important to consider whether participation will reflect similar power dynamics that people with ID have historically faced from positions in authority, which may disempower people and perpetuate discrimination and oppression.

Participation of people with ID through advisory boards, or other forms of patient and public involvement (PPI) is important at different stages of research, and is a common way that people with ID are included and actively involved in research. A major criticism of this type of inclusion is that advisory boards are generally made up of those with mild ID or those from self-advocacy backgrounds. Those with additional needs are not necessarily included in these processes. The disability rights movement slogan ‘nothing about us without us’ is a reminder that people with ID should be included in research. The use of advisory boards, as what sometimes appears to be a tokenistic tick-box exercise, are not enough for research to be described as inclusive, particularly when it has been demonstrated that people with moderate and severe ID are able to engage substantially in research and service development.(6) An ID should be considered a valuable form of diversity and methods should be employed to enable participation of diverse groups. An awareness of intersectionality can be helpful to empower those who face additional disadvantage and those that may be further underserved. This may include people with ID with visual impairments or from minoritised ethnic backgrounds.

People with moderate, severe, or profound ID, with additional communication needs risk being excluded from research when employing methods that do not support them to participate. The dominant ableist narrative, by which researchers select methods that require participants to be physically, cognitively and socially able to discuss topics, actively discriminates and excludes from research the voices who are not able to participate as those without an ID. It also amplifies preconceptions about the needs of people with ID without having sufficient mechanisms in place to understand their lived experiences. Methods of inquiry which do not allow people with ID to be heard perpetuate epistemic injustices. From a human rights perspective, this discrimination is also problematic.

Just as participation within research can strengthen the argument for the provision of just and equitable care to be prioritised; there is an urgent need to involve people with ID and their carers in service development. We would argue that high quality research can form part of the solution for influencing change, for example, large scale qualitative research studies can provide a mechanism by which the health service experiences of people with ID can be seen and understood. It seems contradictory to be working in a health system that on surface level advocates for health equity and holistic, person-centred care yet its research foundations inadequately include or capture the lived experience of people with ID. Should we then therefore be shocked when all aspects of health (mental health, physical health and public health including health promotion and disease prevention) are staggeringly poor? From a social justice view, there lies a responsibility to address the inequity of participation in health research. Incorporating individuals in research in a way which meets their communication needs and allows their voices to be heard may be a good place to start.

Participatory research is a term used to describe research design and methods or approaches that have direct collaborations with those being studied for change. Participatory research has potential to understand the experiences of people with ID and gain insights which may otherwise be lost through less inclusive methods. It is both a method and ideology where an individual’s experience, knowledge and reality is seen as valuable with an awareness of power dynamics between researchers and participants. Participatory methods can be used at all stages of research and have the power of bringing together different forms of knowledge to work towards making change a reality. Examples include photovoice, body-mapping and art-based methods. Evaluating the impact of participatory research in public health research has shown systemic changes, development of unanticipated projects and sustained efforts towards health improvement,(7). Therefore, involving people with ID in research through participatory methods has potential to shape health policy and enable health service failings to be addressed.

High quality research looking at treatment efficacy of common mental health conditions in people with ID is lacking. Randomised controlled trials help understand whether an intervention is successful, though there remain significant concerns around the ethics of randomisation, capacity to consent and their impact on participant recruitment and ethical approvals,(5, 8) disproportionately affecting those with moderate to profound ID. Participatory research however can go a step further and offer insights into the successes or failures of interventions by providing a deeper understanding on how the intervention is lived and experienced by different people and how it can be improved and sustained. This is important when making clinical decisions on evidence-based research. Though it may not be entirely achievable to accurately understand experiences of those with severe or profound ID, participatory approaches along with carer involvement may attempt to reduce the biases of methods that exclude them. A mixed methods approach, which includes a component of participatory methodology may be a more balanced approach to maintaining quality whilst ensuring that research is inclusive.

Access to representative groups and fostering greater involvement of people with ID may require a focus on relationship and trust building and understanding the impact of participation. Utilising accessible venues or environments where individuals feel comfortable is important, in addition to the provision of accessible information in different formats where there are communication, literacy and language barriers.(9) Applying a personalised approach to improve involvement of people with ID and their carers in research is therefore required. This may include the involvement of experienced multidisciplinary professionals and researchers who have experience in working with people with ID, such as speech and language therapists. Significant barriers to participatory methods therefore will include constraints in resources such as funding and time required to develop and implement inclusive projects.

The pertinent questions remain: Have we really learnt from figures illustrating poor health outcomes if research methodology continues to employ methods which do not enable participation? How do we seek to address the barriers to care that contribute to these health outcomes and make real change if the research community does not fully engage and include people with ID? Methods of inquiry which do not fully capture the experiences of people with ID can be harmful. We cannot ‘help and do no harm’ whilst turning a blind eye to the clear injustices that exist in both the clinical and research world. Utilising participatory methods may therefore be a stepping-stone in the right direction, where research starts with inclusivity and supporting individuals to participate. We are yet to see the implementation of radical and sustained changes where researchers actively seek out and incorporate the patient-voice through participatory methods. There needs to be a strong cultural shift to enable discussions on how we can move forward and empower people with ID to participate in research. We propose meeting research participants with ID in a way which removes barriers and employ methods which enables participation in research that involves them and directly impacts them. This perhaps, is not as radical as it seems.

## Data Availability

Data availability is not applicable to this article as no new data were created or analysed in this study
